# Retraction: The Rational Design of Specific Peptide Inhibitor against p38α MAPK at Allosteric-Site: A Therapeutic Modality for HNSCC

**DOI:** 10.1371/journal.pone.0295637

**Published:** 2023-12-08

**Authors:** The PLOS ONE Editors

Following the publication of this article [1], concerns were raised regarding western blot results in Fig 5 and the use of a potentially contaminated cell line. Specifically,

- The p38α panel in Fig 5C and phosphorylated ATF-2 panel in Fig 5D appear to have vertical discontinuities between lanes 1/2 and 2/3 suggested of splicing.
- The p38α panel in Fig 5C of this article [1] appears similar to the p38α panel in Fig 4C of [2]. Additionally, lanes 1 and 3 of this panel appear similar to lanes 1 and 2 of the P38β panel of Fig 4B in [3].
- Lanes 1 and 2 of the phosphorylated ATF-2 panel in Fig 5D of this article [1] appear similar to lanes 1 and 2 of the phosphorylated ATF-2 panel in Fig 4D of [2] and lanes 1 and 3 of the phosphorylated ATF-2 panel in Fig 4B of [3].
- The KB cell line described as an oral cancer cell lines has been shown to be potentially contaminated or a likely derivative of HeLa cervical cancer cell line [4].

The corresponding author stated that all primary data underlying this article remain available. They provided files described as the original raw images underlying the p38α and phosphorylated ATF-2 panels in Fig 5. These images also appeared to have discontinuities between the lanes, and the author’s explanation did not resolve the concerns.

The corresponding author agreed that the p38α and phosphorylated ATF-2 panels in Fig 5 of this article [1] appear similar to the indicated panels in the other articles [2, 3], and they claimed that the similarity was due to the comparable experimental conditions. The editors remain concerned about the level of similarity between images representing different experimental conditions.

The corresponding author acknowledged the concerns regarding potential contamination of the KB cell line; however, they stated that STR profile analysis is not available to confirm the identity of the cells used in this study.

In light of the concerns that question the reliability and integrity of these data, the *PLOS ONE* Editors retract this article.

SD did not agree with the retraction. All other authors either did not respond directly or could not be reached.

Figs 5C and D appear to report previously published material from [2, 3], published in 2012 by Elsevier and John Wiley & Sons, which are not offered under a CC BY license and are therefore excluded from this article’s [1] license. At the time of retraction, the article [1] was republished to note this exclusion in the Fig 5 legend and the article’s copyright statement.
